# Patient activation and psychological coping strategies to manage challenging circumstances during the COVID-19 pandemic in people with kidney disease

**DOI:** 10.1007/s40620-023-01851-1

**Published:** 2024-01-18

**Authors:** Courtney Jane Lightfoot, Thomas James Wilkinson, Naeema Aiyub Patel, Ceri Rhiannon Jones, Alice Caroline Smith

**Affiliations:** 1 Leicester Kidney Lifestyle Team, Department of Health Sciences, University of Leicester, Leicester, LE1 7RH UK; 2 Leicester NIHR Biomedical Research Centre, Leicester, UK; 3 Department of Neuroscience Psychology and Behaviour, University of Leicester, Leicester, UK

**Keywords:** Chronic kidney disease, Patient activation, Self-management, psychological coping, Coping behaviours, COVID-19

## Abstract

**Background:**

Coping with health problems requires some degree of self-management; however, an individual’s ability to self-manage can be threatened during challenging times, such as the COVID-19 pandemic. Exploring differences and changes in psychological well-being and coping strategies between those with low and high patient activation may inform appropriate interventions to support psychological coping.

**Methods:**

People with chronic kidney disease (CKD) (non-dialysis and transplant) were recruited from 11 hospital sites across England between August and December 2020. Participants responded to an online survey study, including the Brief Coping Orientation to Problem Experienced (COPE) Inventory, Depression, Anxiety and Stress Scale (DASS-21), Short Health Anxiety Index (SHAI), and Patient Activation Measure (PAM-13). A follow-up survey was conducted 6–9 months later. Paired *t* tests assessed within-group changes, and chi-squared tests compared coping strategies utilised by low- and high-activated participants. General linear modelling was performed to determine the relationship between patient activation and coping strategies, and covariates.

**Results:**

Two hundred and fourteen participants were recruited (mean age: 60.7, 51% male, mean eGFR: 38.9 ml/min/1.73 m^2^). Low-activated participants were significantly more anxious than high-activated participants (*P* = 0.045). Health anxiety significantly decreased (i.e., got better) for high-activated participants (*P* = 0.016). Higher patient activation scores were associated with greater use of problem-focused strategies (*β* = 0.288, *P* < 0.001). Age (*β* = − 0.174, *P* = 0.012), sex (*β* = 0.188, *P* = 0.004), and education level (*β* = 0.159, *P* = 0.019) significantly predicted use of problem-focused strategies.

**Discussion:**

Those with higher activation had lower levels of anxiety, and more frequently used adaptive coping strategies during the pandemic. Targeted support and interventions may be required for people with CKD to enhance patient activation, encourage more positive adaptive coping strategies, and mitigate maladaptive coping strategies.

**Graphical abstract:**

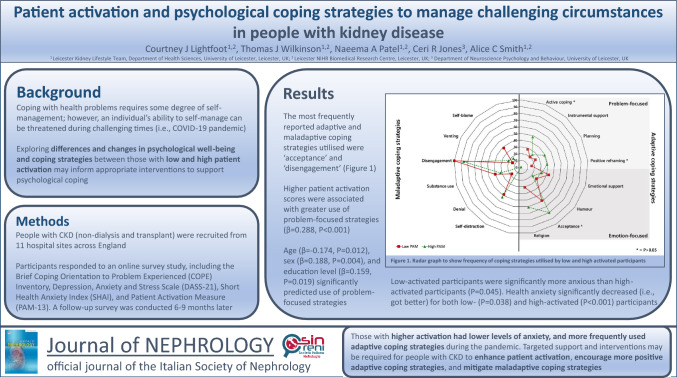

**Supplementary Information:**

The online version contains supplementary material available at 10.1007/s40620-023-01851-1.

## Introduction

Managing long-term conditions, like chronic kidney disease (CKD), and dealing with the associated health and psychosocial problems requires some degree of self-management. Successful self-management involves having the knowledge to understand what to do and why, the skills to be able to perform the required tasks or behaviours, and the confidence to do them—termed patient activation [1]. An individual’s ability to self-manage their health can be threatened during challenging/stressful times, such as the coronavirus disease 2019 (COVID-19) pandemic. The adverse impacts of COVID-19, including drastic changes in day-to-day routines [2] and interruptions to routine healthcare [3, 4], had a profound negative impact on psychological well-being [5], particularly for individuals with long-term conditions like CKD [6, 7].

Cognitive stress appraisal enables individuals to identify if they have the necessary resources to manage stress(ors)—referred to as coping strategies [8, 9]. Lazarus and Folkman’s [10, 11] transactional model of stress and coping is the predominant theoretical model that underpins how individuals appraise stress and how they adapt (or not). Coping strategies (defined as cognitive, emotional, and/or behavioural response(s) to stress [12]) can be divided dichotomously into ‘problem-focused’ (efforts to modify the problem, e.g. active coping, behavioural change) and ‘emotional-focused’ (efforts to manage the emotional distress, e.g. emotional support, denial) dimensions [8, 13], or into ‘approach’ versus ‘avoidant’ coping styles [14, 15]. Non-avoidant adaptive coping, ‘problem-focused’ (e.g. active coping, planning, instrumental support) or ‘emotion-focused’ (e.g. acceptance, positive reframing, emotional support), is when individuals take appropriate precautionary action toward the stressful event for self-protection and can lead to resilience in the face of stress(ors). However, when an individual considers themselves to be inadequately equipped to cope, this can lead to further stress and engagement in maladaptive ‘dysfunctional’ or ‘avoidant’ coping strategies (e.g. self-distraction, behavioural disengagement) [16, 17] and may hurt individuals’ self-interests [18]. Appraisal may prompt adaptive or maladaptive responses, and distinct appraisals work mutually to determine individuals’ responses toward the stressful event [17]. Appropriate information may prompt the implementation of adaptive responses and facilitate protective behaviour, whereas a lack of fear may provoke maladaptive responses and result in dangerous behaviour [19]. Whilst some coping responses may be beneficial for some people in some situations, they may not be beneficial for others or in other situations [20]. A given coping strategy may not be intrinsically maladaptive but may become dysfunctional if it is relied on for long periods when other strategies are more useful [15]. The bipolar coping dimensions are not mutually exclusive and can be applied simultaneously, demonstrating low or high engagement with either the problem or emotions [21].

It could be hypothesised that greater levels of patient activation may have a positive effect on psychological well-being and coping. Exploring this relationship could provide a better understanding of the types of coping strategies used by low- and high-activated individuals, enabling healthcare professionals to offer tailored help and support to patients during ongoing or future stressors which may potentially impact their ability to self-manage. Thus, this study aimed to identify differences in psychological well-being and coping strategies between those with low and high patient activation levels, and to explore the associations between patient activation and coping strategies in people with CKD during the COVID-19 pandemic—a time of potentially challenging circumstances.

## Methods

### Study design and setting

The data presented here were taken from a survey-based longitudinal sub-study of the multi-centre observational DIMENSION-KD study (ISRCTN84422148). In 2020, the DIMENSION-KD study was adapted in response to the developing COVID-19 pandemic. The adapted study aimed to explore the impact of the COVID-19 pandemic on lifestyle determinants and factors associated with living with CKD, healthcare provision, risk perception and coping strategies in people with CKD. The present data collection commenced in August 2020. Participants completed two online surveys. The initial survey consisted of two parts: Part 1 included demographic information, and questions designed to assess participants’ understanding and beliefs of COVID-19 and its impact; Part 2 included validated questionnaires assessing patient activation, health anxiety, and coping strategies. The follow-up survey, completed between May and June 2021, was a condensed version of the initial survey and included the validated questionnaires. Data were collected using Jisc Online Surveys (Bristol, UK). UK COVID-19 restrictions in place at the survey timepoints are detailed in Supplementary Material 1. The study received national research ethical approval by the Leicester Research Ethics Committee (18/EM/0117). All participants provided informed written consent and the study was conducted in accordance with the Declaration of Helsinki.

### Participants

Participants were recruited between August and December 2020 across 11 hospital sites in England, UK. Participants were included if they: (1) had been diagnosed with a kidney condition (CKD stages 1–5 not requiring dialysis (ND-CKD)), or were a kidney transplant recipient; (2) were aged ≥ 18 years; and (3) were able to provide informed consent. Those receiving dialysis were excluded from the study as their treatment and healthcare continued ‘as normal’ during the study period.

### Outcome measures

#### Sociodemographic

Basic self-reported sociodemographic variables, including age, sex, ethnicity, and social deprivation (via postcode), were collected, along with self-reported CKD status and other health conditions, and COVID-19 infection. Participants’ most recent clinical data, including kidney function (estimated glomerular filtration rate, eGFR), cause of disease, haemoglobin, and albumin were extracted from their medical records.

#### Patient activation measure (PAM-13)

The PAM-13 is a 13-item questionnaire designed to assess an individual’s knowledge, skills, and confidence in managing their health(care) [22]. Items are measured on a 4-point Likert scale ranging from “strongly disagree” to “strongly agree”. The PAM-13 is scored from 0 to 100, which correlates to one of four levels. PAM Levels 1 (PAM-13 score: ≤ 47) and 2 (47.1–55.1) indicate lower activation; Levels 3 (55.2–67) and 4 (≥ 67.1) indicate higher activation. The PAM-13 shows good internal consistency and has been validated in CKD [23].

#### Coping orientation to problems experienced inventory (Brief-COPE)

The Brief-COPE is a 28-item questionnaire designed to assess a range of coping responses in relation to a stressful life event [14]. Items are measured on a 4-point Likert scale ranging from “I haven’t been doing this at all” to “I’ve been doing this a lot”. There are 14 scales comprising two items each, with scores ranging from 2 (minimum) to 8 (maximum). The scale can indicate the degree to which the respondent has been engaging in each coping style (higher scores indicate increased utilisation) and can determine one’s primary coping style: problem-focused, emotion-focused, and avoidant. The Brief-COPE has established good internal consistency, reliability, and validity. Although these coping strategies overall cannot be termed as adaptive or maladaptive and are dependent on the context and situation, we have clustered them using the suggested grouping defined previously [24–26]. Adaptive stress-coping included several strategies: religion; active coping; planning; acceptance; positive reframing; instrumental support; emotional support; and humour. Maladaptive stress-coping included several strategies: behavioural disengagement; denial; self-distraction; self-blame; substance use; and venting.

#### Short health anxiety inventory (SHAI)

The SHAI is an 18-item instrument which assesses health anxiety (worry about health, awareness of bodily sensations or changes, feared consequences of having an illness) independently of physical health status [27]. Items are weighted 0–3 and are summed to obtain a total score (0–54), with higher scores indicating increased health anxiety. The SHAI has demonstrated good reliability and validity [27]. The SHAI was developed as a brief screening tool [27] and is widely used by clinicians and researchers assessing health anxiety symptoms across non-clinical, clinical, and medical samples [28].

#### Depression, anxiety and stress scale (DASS-21)

The DASS-21 is a 21-item questionnaire assessing three scales designed to measure the emotional states of depression, anxiety, and stress [29]. Each scale contains 7 items assessed on a 4-point Likert scale ranging from “never” to “almost always”. Scores for each scale are calculated by summing the scores of the relevant items and are summed for a total score (0–120). The DASS-21 has excellent internal consistency and reliability [30]. The DASS is a screening tool designed to assess symptoms of depression, anxiety, or stress, but cannot diagnose them as conditions [31].

### Data analysis

Descriptive and frequency statistics were used to describe participant characteristics, and are presented as mean (standard deviation (SD)), and change-related data are presented as means (95% confidence intervals) unless otherwise stated. Baseline characteristics were compared using independent samples *t* tests. Within-group changes were analysed by paired-sample t-tests or Wilcoxon signed-rank test, as appropriate. Statistical analysis was performed using IBM SPSS 26 software (IBM, Chicago, IL). Statistical significance was accepted as *P* < 0.05.

Participants were categorised into ‘low’ and ‘high’ activation based on their PAM Level. Coping strategies were classed into binary variables to indicate the degree of engagement (i.e., frequently used and not used). A score ≥ 6 indicates that they moderately engaged with the coping strategy (i.e., ‘a medium amount’ or ‘a lot’). Coping strategies were classified into *adaptive* (active coping, information support, positive reframing, planning, emotional support, humour, acceptance, and religion) and *maladaptive* (venting, self-blame, self-distraction, denial, substance use, and behavioural disengagement) coping strategies. Frequency analysis and Chi-squared tests were conducted to compare coping strategies used between high and low-activated participants. General linear models were fitted to determine between-group differences with the change as the dependent variable and the group assignment, age, sex, ethnicity, and CKD stage as covariates.

Data on the impact of COVID-19 on people living with CKD and kidney healthcare provision during the pandemic are reported elsewhere [32–34].

## Results

### Baseline characteristics

A total of 214 participants completed the initial questionnaire (timepoint 1) and were included in the analysis. Seventy-seven (36%) completed it during a period of no restrictions, 48 (22%) during a national lockdown, 36 (17%) when their local area was in Tier 1 (medium restrictions), 31 (15%) in Tier 2 (high restrictions), and 22 (10%) in Tier 3 (very high restrictions). Of these, 109 (51%) were male and 197 (92%) were White British; the mean age was 60.7 years (SD 14.1 range 18–89). One hundred and twenty participants (56%) were kidney transplant recipients. The mean eGFR for ND-CKD participants was 38.9 (SD 23.8) ml/min/1.73 m^2^. Of the 214 who completed the initial survey, 93 (43%) completed the follow-up survey (timepoint 2) and were included in a cohort assessing changes in mental health status and coping strategies employed between timepoints. Of these, 49 (53%) were male and 87 (94%) were White British; the mean age was 63.9 years (SD 11.5 range 18–89). 50 participants (54%) were kidney transplant recipients. Participant characteristics are detailed in Table 1.

**Table 1:** Participant characteristics

	Timepoint 1 (*n* = 214)	Change cohort (*n* = 93)
Age, years	60.7 (14.1)	63.9 (11.5)
Sex, male *n* (%)	109 (51%)	49 (53%)
Ethnicity		
White British, *n* (%)	197 (92%)	87 (94%)
South Asian, *n* (%)	7 (3%)	2 (2%)
Other, *n* (%)	10 (5%)	4 (4%)
CKD stage		
NDD, *n* (%)	94 (44%)	43 (46%)
Mean eGFR, ml/min/1.73 m^2^	38.9 (23.8)	35.3 (22.5)
TX, *n* (%)	120 (56%)	50 (54%)
Haemoglobin (g/L)		129.5 (18.5)
Albumin (g/L)		41.3 (4.4)
Comorbidities		
Type 2 Diabetes, *n* (%)	39 (18%)	21 (23%)
Hypertension, *n* (%)	168 (79%)	74 (80%)
CVD, *n* (%)	66 (31%)	31(33%)
Depression, anxiety or other mental health problems, *n* (%)	43 (20%)	14 (15%)
BMI, (kg/m^2^)	28.56 (6.95)	28.8 (7.8)
PAM score	66.71 (14.59)	67.9 (14.7)
PAM level		
Level 1, *n* (%)	16 (7%)	3 (3%)
Level 2, *n* (%)	34 (16%)	17 (18%)
Level 3, *n* (%)	104 (49%)	45 (48%)
Level 4, *n* (%)	60 (28%)	28 (30%)

NB. Data shown as mean (standard deviation) unless otherwise stated

*CKD* chronic kidney disease, *NDD* non-dialysis dependent, *TX* transplant, *BMI* body mass index, *CVD* cardiovascular disease, *PAM* Patient Activation Measure

Forty-three (20%) individuals reported depression, anxiety, or other mental health problems. The mean scores for anxiety, depression, and stress were 2.75 (± 3.38), 4.75 (± 4.22), and 4.89 (± 3.90), respectively. The mean SHAI score was 13.0 (± 6.6). The mean PAM score was 66.7 (± 14.6). Kidney transplant recipients had significantly higher PAM scores (70.0 ± 13.7) compared to ND-CKD participants (62.5 ± 14.7) (*P* < 0.001). Scores are displayed in Table 2.

**Table 2:** Anxiety, depression, stress, health anxiety and PAM-13 scores of low and high activated participants

Timepoint 1					
	Total (*n* = 214)	Low PAM-13 (*N* = 50)	High PAM-13 (*N* = 164)	Difference between groups	Effect size (Cohen’s d)
Anxiety	2.75 (3.13)	3.53 (3.44)	2.51 (2.99)	*P* = 0.045	*d* = 0.329
Depression	4.75 (4.22)	5.54 (5.13)	4.49 (3.87)	*P* = 0.189	*d* = 0.248
Stress	4.89 (3.90)	5.40 (4.66)	4.73 (3.63)	*P* = 0.287	*d* = 0.173
Total DASS score	12.12 (10.04)	14.16 (12.13)	11.45 (9.21)	*P* = 0.100	*d* = 0.271
Health anxiety	12.99 (6.64)	14.50 (7.79)	12.54 (6.21)	*P* = 0.132	*d* = 0.297
PAM-13 score	66.71 (14.59)	48.93 (4.54)	72.13 (12.06)	*P* < 0.001	*d* = 2.148

NB. Data shown as mean (standard deviation) unless otherwise stated

*DASS* depression, anxiety, stress scale, *PAM-13* patient activation measure

^*^P < 0.05

### COVID-19 infection rates

At timepoint 1, 2% (*n* = 11) self-reported having a positive COVID-19 test result, and 3% (n = 16) suspected having COVID-19 but this was not confirmed by a test result. Eighty-six percent reported not being infected and 9% were unsure. At timepoint 2, 3% (*n* = 8) reported having a positive test result and 2% (*n* = 5) suspected having COVID-19. Forty percent (*n* = 93) reported testing and having a negative result, and 53% (*n* = 123) stated not suspecting that they had COVID-19 nor experienced any symptoms. Of those who reported having COVID-19 (*n* = 13), 92% reported staying at home, with one individual (8%) admitted to hospital. Fifty percent reported having mild symptoms, 33% moderate, and 17% severe symptoms. Sixty-two percent reported experiencing symptoms for less than two weeks, 23% between two weeks and a month, and 15% for more than a month.

### Levels of anxiety, depression, stress, and health anxiety

Table 2 shows the levels of anxiety, depression, stress, and health anxiety of low- and high-activated participants at both timepoints. Significant differences were observed between anxiety scores of low- and high-activated participants at timepoint 1 (*P* = 0.045). No differences were observed in depression, stress, or health anxiety scores between activation groups, nor between kidney transplant recipients and ND-CKD participants.

### Coping strategies utilised

**Fig. 1:**
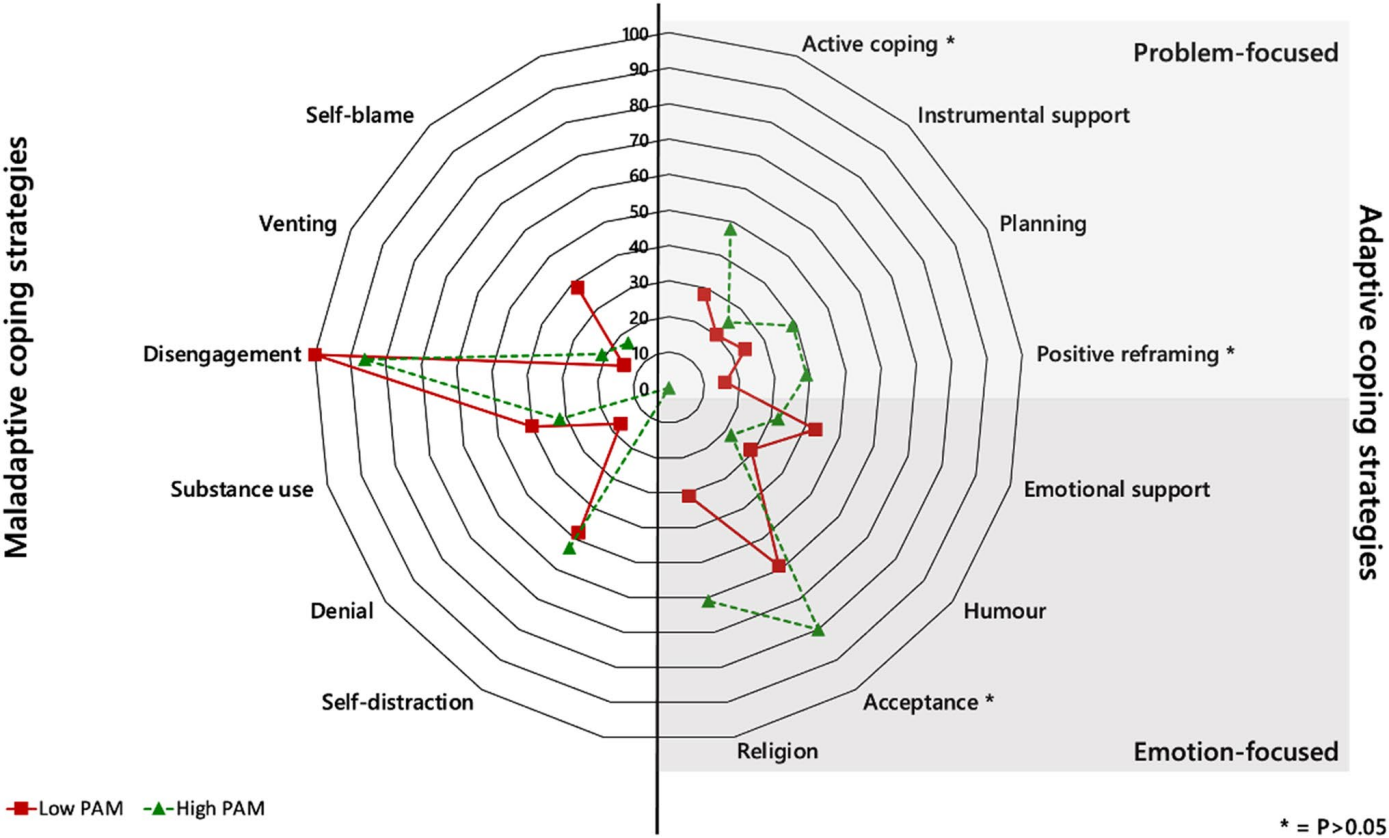
Radar graph to show frequency of coping strategies utilised by low and high activated participants

The proportion of low and high-activated participants who reported using each coping strategy is displayed in Fig. 1 (data are available in Supplementary Material 2).

‘Acceptance’ was the most frequently reported adaptive coping strategy utilised by both low (59%) and high (80%) activated participants. A significantly greater proportion of high-activated participants reported using adaptive coping strategies: ‘active coping’ (*P* = 0.037), ‘positive reframing’ (*P* = 0.026), and ‘acceptance’ (*P* = 0.007). A significantly greater proportion of kidney transplant recipients used positive reframing (*P* = 0.038) compared to ND-CKD.

‘Disengagement’ was the most frequently reported maladaptive coping strategy utilised, with 100% of low-activated and 86% of high-activated participants using it. ‘Self-distraction’ (53% and 48%) and ‘substance use’ (32% and 40%) were the next most reported maladaptive coping strategies by both high- and low-activated participants, respectively. No significant differences were observed in the maladaptive coping strategies used.

### Relationship between patient activation and coping strategies

Table 3 displays the mean scores for each coping strategy. Higher patient activation scores were associated with greater use of problem-focused strategies (*β* = 0.288, *P* < 0.001), including active coping (*β* = 0.319, *P* < 0.001), positive reframing (*β* = 0.364, *P* < 0.001), planning (*β* = 0.234, *P* < 0.001), and acceptance (*β* = 0.192, *P* = 0.035).

**Table 3:** Coping strategy scores of low and high activated participants

Timepoint 1					
	Total (*n* = 214)	Low PAM-13 (*N* = 50)	High PAM-13 (*N* = 164)	Difference between groups	Effect size (Cohen’s d)
Self distraction	5.60 (1.14)	5.38 (1.02)	5.65 (1.16)	*P* = 0.326	*d* = 0.237
Active coping	5.32 (1.18)	4.69 (0.89)	5.48 (1.19)	*P* = 0.001*	*d* = 0.697
Denial	4.50 (0.67)	4.83 (0.75)	4.17 (0.41)	*P* = 0.086	*d* = 1.101
Substance use	4.83 (1.13)	4.80 (1.10)	4.84 (1.17)	*P* = 0.341	*d* = 0.036
Emotional support	5.15 (1.17)	5.25 (1.14)	5.12 (1.18)	*P* = 0.601	*d* = 0.111
Instrumental support	4.81 (1.19)	4.60 (1.07)	4.84 (1.21)	*P* = 0.552	*d* = 0.207
Disengagement	4.88 (1.09)	4.44 (1.01)	5.13 (1.09)	*P* = 0.138	*d* = 0.640
Venting	4.78 (1.03)	4.54 (0.78)	4.88 (1.11)	*P* = 0.319	*d* = 0.330
Positive reframing	5.18 (1.17)	4.64 (0.76)	5.31 (1.21)	*P* = 0.009*	*d* = 0.588
Humour	4.79 (0.98)	4.83 (1.17)	4.77 (0.92)	*P* = 0.805	*d* = 0.058
Acceptance	6.43 (1.24)	6.02 (1.25)	6.54 (1.22)	*P* = 0.018*	*d* = 0.418
Religion	5.73 (1.61)	5.23 (1.54)	5.89 (1.62)	*P* = 0.203	*d* = 0.414
Self-blame	4.84 (1.24)	5.13 (1.13)	4.74 (1.29)	*P* = 0.458	*d* = 0.309
Planning	5.23 (1.11)	4.76 (0.83)	5.34 (1.15)	*P* = 0.019*	*d* = 0.530
Problem-focused	13.75 (6.38)	11.15 (5.40)	14.52 (6.45)	*P* = 0.001*	*d* = 0.542
Emotion-focused	17.63 (6.67)	17.22 (7.18)	17.75 (6.53)	*P* = 0.631	*d* = 0.398
Avoidant	7.22 (3.81)	7.05 (4.06)	7.27 (3.74)	*P* = 0.728	*d* = 0.396

*NB* Data shown as mean (standard deviation) unless otherwise stated, *DASS* Depression, Anxiety, Stress Scale, *PAM-13* Patient Activation Measure

*P<0.05

### Factors predicting coping strategies used

Being younger significantly predicted use of problem-focused (*β* = 0.174, *P* = 0.012), emotion-focused (*β* = 0.153, *P* = 0.039), and avoidant coping strategies (*β* = 0.226, *P* = 0.002). Being female significantly predicted avoidant coping (*β* = 0.174, *P* = 0.016). Higher levels of education level significantly predicted the use of problem-focused strategies (*β* = 0.159, *P* = 0.019), active coping (*B* = 0.178, *P* = 0.036), instrumental support (*β* = 0.280, *P* = 0.044), and planning (*β* = 0.180, *P* = 0.048). Lower levels of education significantly predicted the use of self-distraction (*β* = 0.260, *P* = 0.008) and positive reframing (*β* = 0.174, *P* = 0.029). Being non-White significantly predicted the use of substance use (*β* = 0.513, *P* = 0.040), behavioural disengagement (*β* = 0.605, *P* = 0.033), and positive reframing (*β* = 0.171, *P* = 0,037). Decreased social deprivation significantly predicted the use of positive reframing (*β* = 0.174, *P* = 0.029).

### Changes in mental health status

Changes in anxiety, depression, stress, and health anxiety scores between low- and high-activated participants are displayed in Table 4. Anxiety, depression, and stress scores (DASS-21) did not significantly change between timepoints for either low- or high-activated participants. Health anxiety scores (SHAI) significantly decreased (i.e., got better) between timepoints for high-activated participants (*P* = 0.016), but not for low-activated participants, with no significant difference for the change in health anxiety scores between the two groups.

**Table 4:** Changes between timepoint 1 (August and December 2020) and timepoint 2 (May and June 2021) for low and high PAM-13 groups

	Low PAM-13 (*n* = 20)	High PAM-13 (*n* = 73)	Difference between groups
Anxiety			
Timepoint 1	3.35 (3.60)	2.20 (2.75)	*P* = 0.110
Timepoint 2	2.65 (3.53)	2.56 (2.72)	*P* = 0.914
Change	− 0.70 (1.72) (95% CI − 1.50 to 0.10) *P* = 0.085 *d* = 3.561	0.36 (2.41) (95% CI − 0.20 to 0.94) *P* = 0.205 *d* = 3.195	1.06 (95% CI − 0.08 to 2.21) *P* = 0.068 *d* = 0.536
Depression			
Timepoint 1	4.80 (5.26)	3.94 (3.83)	*P* = 0.269
Timepoint 2	4.45 (4.91)	3.88 (3.90)	*P* = 0.444
Change	− 0.35 (3.86) (95% CI − 2.15 to 1.45) *P* = 0.689 *d* = 1.709	− 0.06 (3.77) (95% CI − 0.96 to 0.85) *P* = 0.899 *d* = 2.333	0.29 (95% CI − 1.62 to 2.20) *P* = 0.693 *d* = 0.252
Stress			
Timepoint 1	5.00 (4.93)	4.29 (3.39)	*P* = 0.363
Timepoint 2	4.25 (4.42)	4.35 (3.55)	*P* = 0.982
Change	− 0.75 (2.73) (95% CI − 2.02 to 0.53) *P* = 0.234 *d* = 2.232	0.06 (3.35) (95% CI − 0.73 to 0.84) *P* = 0.888 *d* = 2.257	0.81 (95% CI − 0.82 to 2.43) *P* = 0.326 *d* = 0.265
Health anxiety			
Timepoint 1	13.29 (7.07)	12.09 (5.85)	*P* = 0.414
Timepoint 2	12.12 (8.75)	10.94 (6.35)	*P* = 0.468
Change	− 1.18 (4.77) (95% CI − 3.63 to 1.28) *P* = 0.325 *d* = 0.246	− 1.15 (3.80) (95% CI − 2.17 to 0.47) *P* = 0.016 *d* = 0.303	0.03 (95% CI − 2.15 to 2.20) *P* = 0.982 *d* = 0.006
PAM-13 score			
Timepoint 1	50.25 (5.02)	72.72 (12.64)	*P* < 0.001
Timepoint 2	54.41 (13.66)	69.28 (16.49)	*P* < 0.001
Change	4.17 (12.37) (95% CI − 1.62 to 9.96) *P* = 0.149 *d* = 0.337	− 3.44 (13.24) (95% CI − 6.53 to 0.35) *P* = 0.030 *d* = 0.260	− 7.61 (95% CI − 14.15 to − 1.06) *P* = 0.023 *d* = 0.582

NB. Data shown as mean (standard deviation) unless otherwise stated. Effect size: (Cohen’s d)

*DASS* depression, anxiety, stress scale, *PAM* patient activation measure

*P < 0.05

### Changes in coping strategies utilised

At timepoint 2, a significantly lower proportion of low-activated participants reported using emotional support (*P* = 0.003), humour (*P* = 0.035), acceptance (*P* = 0.039), and religion (*P* = 0.025) as coping strategies than at timepoint 1. For high-activated individuals, a significantly lower proportion used self-distraction (*P* = 0.001), active coping (*P* = 0.011), emotional support (*P* < 0.001), instrumental support (*P* = 0.005), positive reframing (*P* − 0.007), humour (*P* = 0.032), and religion (*P* = 0.001) as coping strategies at timepoint 2 compared to timepoint 1. In addition, the reported use of emotional-focused (*P* = 0.001) and avoidant (*P* = 0.012) coping strategies was lower at timepoint 2 by high-activated individuals.

### Changes in patient activation

Changes in PAM-13 scores between low- and high-activated participants are displayed in Table 4. PAM-13 scores for high-activated participants significantly decreased between timepoints (*P* = 0.030). No significant change was observed in low-activated individuals. There was a significant difference in the change of PAM score between low- and high-activated individuals (*P* = 0.023).

## Discussion

Our findings showed that low-activated participants had significantly higher levels of anxiety during the COVID-19 pandemic compared to high-activated participants. They also had greater levels of depression, stress, and higher levels of health-related anxiety when compared to high-activated participants, although not statistically significant. Between timepoints, no significant differences between low- and high-activated participants for anxiety, depression, stress, or health anxiety were observed. Only high-activated participants had a significant change across timepoints for health anxiety. Whilst the most common adaptive (‘acceptance’) and maladaptive (‘behavioural disengagement’) coping strategies used were similar, a greater proportion of high-activated individuals used adaptive coping strategies and low-activated individuals used maladaptive ones. Higher patient activation was associated with significantly greater use of problem-focused strategies, with age, sex, and education level significantly predicting their usage.

The findings from this study can be interpreted in the context of coping theories. When an individual is faced with stressful situations, like those experienced during the pandemic, they will appraise the stressor and utilise resources to enable stress reduction [8]; this can involve applying strategies to the given situation to manage, altering the problem causing the distress (‘problem-focused’) and/or regulating emotional responses to the problem (‘emotion-focused’) [8]. Given that patient activation considers an individual’s ability to maintain their behaviours in times of stress [35], it is perhaps unsurprising that we found that high-activated individuals more frequently reported utilising problem-focused coping strategies.

The results suggest that a high proportion of individuals, regardless of activation level, exhibited behavioural disengagement. Experiential avoidance (i.e., avoiding thinking about and reducing efforts to deal with stress(ors)) is a common response that enables temporary relief; whilst this may elevate distress in the short-term, it can prolong or amplify distress long-term [36]. If avoidance becomes an inflexible pattern of behaviour, this can affect an individual’s functioning across different areas of their life and can lead to significant long-term health effects [37], including depression and anxiety [38]. Adoption of new coping strategies, particularly problem-focused as opposed to emotion-focused, can lead to improvements in mental health [39, 40], and can help people move from a place of avoidance to more changeable domains (i.e., overt behaviour) to enact a process of adjustment.

The frequency, and type, of coping strategies used changed between timepoints. This supports previous evidence which suggests that coping changes over time [41]. Coping is a dynamic and transactional process whereby the individual considers the situation through a complex evaluation process [42]; the individual’s perception of the significance of the situation in relation to their personal values, beliefs, or intentions (known as primary appraisal), and the examination of the resources/coping options available to reduce negative arousal and increase positive outcomes [8]. Events related to the COVID-19 pandemic may have affected individuals’ coping resources and their usual psychological responses [41, 43]. Stress experienced can be highly individual and dependent on numerous factors—the way individuals cope not only relates to socio-demographic/psychosocial predictors of coping but also prior adversities and personality traits [41]. There is not one coping strategy that is uniformly better for managing stress [36]; those who are better able to regulate their emotions and engage more positively may be more likely to use a range of coping strategies [44]. Individuals who engage in a greater number of positive coping strategies have a greater sense of control, level of acceptance, and ability to adjust/adapt their coping responses [44], and higher levels of resilience [45]. Thus, it is perhaps unsurprising that our findings highlight that high-activated individuals used a greater number of adaptive coping strategies.

Worrying, regardless of activation level, a third of individuals reported substance use as a form of coping during the pandemic (e.g., use of alcohol or other drugs to help one feel better and get through it). This finding is somewhat concerning, but not uncommon. Several studies conducted during the COVID-19 pandemic have reported increased alcohol consumption compared to consumption rates before the COVID-19 pandemic [46, 47], as a result of increased stress and boredom [47]. Research has shown increased engagement in binge drinking and extreme binge drinking during COVID-19 [47]. A qualitative study exploring coping strategies employed by individuals during the pandemic, identified the consumption of alcohol as a theme, with participants reporting drinking alcohol to cope and survive [48]. The consumption of alcohol during the pandemic is associated with younger age, more children at home, non-healthcare workers, and being unemployed as a result of COVID-19 [49]. Consuming alcohol as a form of coping, or engaging in other maladaptive coping strategies, could be a risk for developing further health problems [48].

Like Chen et al. [50], but in contrast to others [41], we found that younger adults were more likely to engage with both problem-focused (e.g. planning, active coping) and emotion-focused (e.g. acceptance) strategies. Like others [41], we found that females were more likely to use both avoidant and active coping strategies; this may be a consequence of experiencing greater levels of stress [51, 52]. Similar to other studies [53, 54], we found ethnicity to be associated with the use of religion as a form of coping; when faced with stressful situations, individuals from ethnic groups (e.g. Black, Asian) are more likely to use religion [53, 54]. Socio-demographic characteristics may be an indicator of those who may benefit from targeted interventions and additional support.

Whilst these data were collected during the COVID-19 pandemic, the findings can apply to other challenging situations/periods that may potentially impact an individual’s ability to self-manage. People with CKD experience numerous stressful events during their lives that evoke coping responses, including CKD-related ones (e.g., initial diagnosis, disease progression, transition of treatment) alongside life events (e.g., births, deaths). People with CKD use more maladaptive coping strategies [55], which are associated with poorer illness perceptions and increased psychological distress [56]. Increasing active engagement, self-efficacy, self-regulation, and patient activation [57] could help individuals develop more adaptive coping strategies and reduce psychological distress [58].

Assessing the patient’s activation level may provide an understanding of the coping strategies they may employ. Delivering appropriate tailored help and support, focusing on fostering/developing activation and positive coping, could provide individuals with an armoury or toolbox of adaptive coping strategies that can be applied during challenging circumstances. Tailored, psychotherapeutic and cognitive behavioural interventions, delivered by psychologists and other mental health professionals, can support reductions in psychological distress by challenging distressing beliefs or cognitions, and improving psychological adaptation by emotion regulation to facilitate adapting coping responses to a perceived threat [59, 60]. Whilst the provision of psychosocial interventions for people with CKD is variable, the UK’s Renal Service Transformation Programme has recently gained consensus and provided recommendations on the most appropriate kidney-specific psychosocial management for people living with CKD [61]. One recommendation includes appropriate referral to relevant services (e.g., psychology, counselling or psychotherapy, social work or liaison psychiatry) to support those who have been identified as having psychosocial needs. Targeted interventions for those with less developed coping, including disadvantaged groups (e.g., female, older, non-White, less educated, increased social deprivation), will likely have the greatest impact.

Our study is strengthened by the use of validated questionnaires to assess patient activation and coping strategies utilisation. Whilst we included both ND-CKD patients and kidney transplant recipients, our sample was fairly homogeneous (e.g., older, White ethnicity). We were reliant on self-reported health status and had limited clinical data to verify the self-reported data; however, the comorbidities reported are representative of the general CKD population, so likely that our sample size is generalizable. The low response rate for the follow-up survey may be a result of the increased distribution of surveys during the COVID-19 pandemic which resulted in survey fatigue, reduced response rates, and data collection quality [62]. Despite this, participant characteristics of those who completed the follow-up survey were similar to those who completed the initial survey. The questionnaires used in this analysis were delivered as part of a larger survey and the response rate or completion of the questionnaires reported here might have been greater if they were delivered on their own because the larger survey may have fatigued respondents. The level of patient activation was slightly, but not alarmingly, higher in this cohort than reported in other non-dialysis CKD studies [63–66]. Like other studies, patient activation was higher in our study than in data collected routinely within clinical practice where a smaller proportion of patients had high levels of activation and a greater proportion had Level 1 activation [67]. Due to the pandemic, the study was conducted entirely online which could have resulted in digital exclusion, and those who are not online (typically older, less affluent, with limited education) may be under-represented. As these factors influenced coping strategies utilised, the inclusion of these individuals in future work exploring coping strategies would be advantageous.

## Conclusion

High-activated individuals had lower levels of stress, anxiety, and depression, and more frequently used adaptive coping strategies. Increasing patient activation has the potential to increase skills and confidence when dealing with difficult/challenging situations, adoption of more problem-focused coping strategies, and adaptation of coping response(s). Individuals with lower levels of activation, and at risk of engaging with more avoidant coping strategies, may need targeted support and interventions that enhance patient activation, cognitive flexibility, and reappraisal to strengthen positive coping strategies and mitigate maladaptive coping strategies.

### Supplementary Information

Below is the link to the electronic supplementary material.

Supplementary file1 (DOCX 17 KB)

Supplementary file2 (DOCX 33 KB)

## Data Availability

The data that support the findings of this study are available from the corresponding author upon reasonable request.
