# Supplementary material for: Patient activation and psychological coping strategies to manage challenging circumstances during the COVID-19 pandemic in people with kidney disease
Source: J Nephrol. 2024 Jan 18;37(2):353–64. doi: 10.1007/s40620-023-01851-1 (PMC11043035; doi:10.1007/s40620-023-01851-1)
Supplement: Supplementary file 1 — Supplementary file1 (DOCX 17 KB) [file 40620_2023_1851_MOESM1_ESM.docx]

**Supplementary material**

**Supplementary Material 1**

**UK restrictions**. More detailed and further information can be found:

- <https://www.instituteforgovernment.org.uk/sites/default/files/timeline-lockdown-web.pdf>
- <https://www.instituteforgovernment.org.uk/sites/default/files/timeline-coronavirus-lockdown-december-2021.pdf>

**2020**

**August** Lockdown restrictions eased further, including reopening indoor theatres, bowling alleys and soft play

**September** ‘Rule of six’ – indoor and outdoor social gatherings above six banned in England. Return to working from home and 10pm curfew for hospitality sector

**October** New three-tier system: Tier 1 = medium; Tier 2 = high; Tier 3 = very high

<https://www.ageuk.org.uk/information-advice/coronavirus/coronavirus-guidance/local-lockdown-tiers/>

**November** Second lockdown

**December** End of second lockdown. Introduction of Tier 4: ‘Stay at Home’ alert level

**2021**

**May - July** Limit of 30 people allowed to mix outdoors. ‘Rule of six’ or two households allowed for indoor gatherings. Opening of indoor venues.

**June** Acceleration of vaccination programme

**Supplementary Material 2**

Data for Radar graph to show frequency of coping strategies utilised by low and high activated participants

|  | All  (n=214) | Low PAM  (n=50) | High PAM  (n=164) | P |
| --- | --- | --- | --- | --- |
| Self-distraction | 53% | 48% | 53% | 0.654 |
| Active coping | 45% | 28% | 48% | 0.045 |
| Denial | 8% | 17% | 0% | 0.296 |
| Substance use | 33% | 40% | 32% | 0.722 |
| Emotional support | 35% | 43% | 32% | 0.285 |
| Instrumental support | 25% | 20% | 25% | 0.735 |
| Disengagement | 88% | 100% | 86% | 0.686 |
| Venting | 21% | 15% | 21% | 0.654 |
| Positive reframing | 38% | 16% | 39% | 0.031 |
| Humour | 28% | 29% | 22% | 0.497 |
| Acceptance | 76% | 59% | 80% | 0.006 |
| Religion | 53% | 31% | 61% | 0.064 |
| Self-blame | 22% | 38% | 17% | 0.241 |
| Planning | 37% | 24% | 39% | 0.169 |
